# CT-Derived Body Composition and Diet Quality in Kidney Transplant Recipients: A Single-Center Retrospective Cross-Sectional Study

**DOI:** 10.3390/medicina62030550

**Published:** 2026-03-16

**Authors:** Oktay Bagdatoglu, Pinar Ulubasoglu, Emin Rencber, Murathan Koksal, Omer Iloglu, Mine Sebnem Karakan

**Affiliations:** 1 Department of Nephrology, Ankara Bilkent City Hospital, Ministry of Health, Ankara 06800, Turkey; ranip35@gmail.com; 2 Department of Public Health, Hitit University Corum Erol Olcok Training and Research Hospital, Corum 19040, Turkey; dr.emirencber@gmail.com; 3 Department of Radiology, Ankara Bilkent City Hospital, Ministry of Health, Ankara 06800, Turkey; murathankoksal@gmail.com (M.K.); omeriloglu17@gmail.com (O.I.); 4 Department of Nephrology, Faculty of Medicine, Ankara Yildirim Beyazit University, Ankara 06800, Turkey; sebnemkarakan@gmail.com

**Keywords:** kidney transplant recipients, retrospective observational study, diet quality, myosteatosis, body composition

## Abstract

*Introduction/Objectives:* Body composition changes and diet quality may contribute to metabolic complications and graft outcomes after kidney transplantation. We evaluated the relationships between diet quality and CT-derived body composition components (skeletal muscle mass, muscle quality/myosteatosis, and visceral adiposity) and explored their associations with metabolic markers and graft function. *Materials and Methods:* In this single-center retrospective cross-sectional study, we included 161 adult first kidney transplant recipients (KTRs) with a functioning graft and ≥12 months of follow-up. Body composition was quantified on routine abdominal CT at the L3 level using skeletal muscle index (SMI), mean muscle attenuation (Hounsfield units) for myosteatosis, and visceral adipose tissue area (VAT). Diet quality was scored using the Revised Diet Quality Index (DQI-R). Graft function was followed with creatinine-based estimated glomerular filtration rate (eGFR) calculated by the CKD-EPI equation. *Results*: Mean age was 45.7 ± 13.2 years and 58% were men. The prevalence of low muscle mass was 26.0%, myosteatosis 73.5%, and visceral obesity (VAT ≥ 100 cm^2^) 45.6%. No participant had “good” diet quality; 48.4% had poor diet quality. DQI-R showed a weak positive correlation with SMI (r = 0.157; *p* = 0.047) but was not significantly related to VAT, subcutaneous adipose tissue (SAT), Kidney transplant recipient (VSR) or myosteatosis. In multivariable models, age and VAT were associated with HbA1c, whereas body composition and diet quality variables were not independent predictors of eGFR. Myosteatosis was independently associated with older age. *Conclusions*: Visceral adiposity and impaired muscle quality frequently clustered and were linked to metabolic status. These findings support post-transplant follow-up strategies that go beyond BMI and integrate body composition and nutritional assessment within a multidisciplinary care model.

## 1. Introduction

Kidney transplantation is the most effective treatment for end-stage kidney disease and substantially improves survival and quality of life [1]. As post-transplant survival improves, attention has increasingly shifted toward managing long-term complications, particularly metabolic disorders that can compromise graft and patient outcomes. Nephrology care is increasingly adopting integrated chronic care models that emphasize prevention, nutritional support, body composition monitoring, and multidisciplinary collaboration (e.g., nephrologists, dietitians, transplant nurses) [2]. However, metabolic complications such as weight gain, obesity, hypertension, dyslipidemia, and post-transplant diabetes mellitus (PTDM) are common in the post-transplant period. Approximately half of recipients experience clinically relevant weight gain within the first year, and obesity prevalence may exceed 30% among in kidney transplant recipients (KTRs) [3,4,5].

Although body mass index (BMI) is commonly used to assess adiposity, it does not fully capture fat distribution or metabolic impact. In contrast, visceral adipose tissue (VAT) is strongly linked to insulin resistance, dyslipidemia, systemic inflammation, and cardiovascular complications, independent of BMI [6,7]. As such, visceral adiposity may provide superior risk stratification in KTRs a population characterized by altered body composition. Therefore, reliance on body weight or BMI alone may overlook clinically relevant alterations in body composition. In this context, changes in skeletal muscle mass, muscle quality, and fat distribution may provide additional clinical information. Monitoring body composition (the distribution of fat and muscle compartments) after transplantation may help to better characterize metabolic risk and support graft health.

Sarcopenia is characterized by progressive loss of skeletal muscle mass and strength due to aging or chronic disease. It is frequently observed in chronic kidney disease (CKD) and in kidney transplant candidates, and it is often considered part of the frailty phenotype [8,9,10]. In KTRs, reported sarcopenia prevalence varies widely (3.7–72%), largely due to heterogeneity in diagnostic methods and thresholds. A recent meta-analysis reported a pooled prevalence of approximately 26% in this population [10,11,12]. While some studies suggest that sarcopenia may be associated with poorer graft and patient outcomes, others did not confirm this association [10,13,14,15].

Myosteatosis refers to lipid infiltration within skeletal muscle and is commonly operationalized as reduced muscle attenuation on computed tomography (CT) (Hounsfield units) [15]. It has been reported in CKD and KTRs, with older age, longer dialysis exposure, and obesity proposed as contributing factors [16]. A prevalence of myosteatosis of approximately 24% has been reported in KTRs [17]. Clinically, myosteatosis may capture risk beyond muscle quantity. For example, one study suggested that low muscle attenuation, rather than low muscle area alone, was an independent predictor of mortality [17]. In addition, meta-analytic data indicate that myosteatosis may more than double the risk of graft loss and patient mortality [18].

Obesity is another frequent problem after kidney transplantation. Although obesity is commonly defined as BMI ≥ 30 kg/m^2^, BMI does not adequately reflect fat distribution [5,19]. Visceral adiposity is closely linked to insulin resistance, dyslipidemia, and cardiovascular risk [20]. Accordingly, pre-transplant CT-derived visceral fat has been reported to predict post-transplant diabetes more strongly than BMI [19]. Dietary patterns and overall diet quality may also influence post-transplant outcomes [21,22]. Appetite and intake often increase after transplantation due to relaxation of dialysis-related restrictions and the metabolic effects of immunosuppressive therapy [23]. In this setting, diet quality may be critical for long-term metabolic risk reduction [24].

The primary objective of this study was to assess the association between CT-derived body composition measures and graft function in KTRs. Secondary objectives included evaluating relationships with metabolic parameters and diet quality.

## 2. Materials and Methods

### 2.1. Study Design and Participant Selection

**Figure 1:**
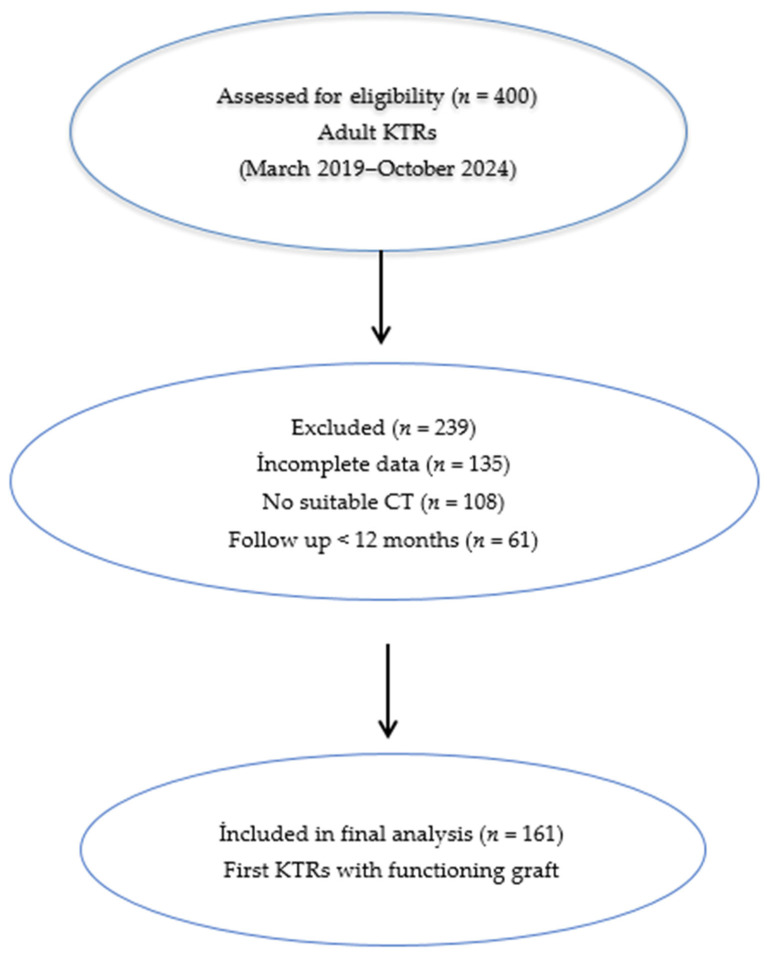
Study flow diagram. Flow diagram illustrating participant selection, exclusion criteria, and the final study population included in the analysis.

This study was designed as a single-center, retrospective observational, cross-sectional analysis and is reported in accordance with the Strengthening the Reporting of Observational Studies in Epidemiology (STROBE) statement. Adult KTRs followed in the transplant outpatient clinic between March 2019 and October 2024 were screened, and a total of 161 eligible first KTRs with a functioning graft and regular follow-up of at least 12 months after transplantation were included in the final analysis. A flow diagram illustrating participant selection, exclusions, and the final analyzed cohort is presented in Figure 1.

*Inclusion criteria:*

- *Age ≥ 18 years*
- *First kidney transplantation*
- *Functioning graft for ≥12 months*
- *Availability of abdominal CT imaging*

*Exclusion criteria:*

- *Multi-organ transplantation*
- *Graft loss or death within first year*
- *Return to dialysis*
- *Active malignancy*
- *Acute infection or hospitalization at index date*
- *Missing key imaging or laboratory data*

The study was approved by the Ankara Bilkent City Hospital Ethics Committee (TABED 2-5-1042) and conducted in accordance with the Declaration of Helsinki. Because this was a retrospective chart review, informed consent was waived by the Ethics Committee.

### 2.2. Basic Information on the Study Population

Demographic and clinical data (age, sex, donor type (living vs. deceased), time since transplantation, and body weight and BMI values before transplantation and at the most recent follow-up visit) were collected retrospectively from medical records. BMI was calculated as weight (kg) divided by height squared (m^2^), and obesity was defined as BMI ≥ 30 kg/m^2^.

### 2.3. Abdominal CT Imaging

Previously acquired abdominal CT scans obtained during routine post-transplant clinical care were reviewed retrospectively; no additional imaging was performed for research purposes. The CT acquisition date was considered the index time point. Diet quality and laboratory data were extracted from the clinical evaluation closest to the index date (e.g., within ±30 days). Abdominal CT images were obtained in the Radiology Department using a 128-detector multi-slice CT scanner (General Electric Revolution Evo 128, Milwaukee, WI, USA). Reported acquisition parameters were: collimation 2 mm, slice thickness 2 mm, rotation time 0.6 s, pitch 1, field of view 40 cm, 120 kV, and 200–400 mA. Body composition analyses were performed on radiology workstations using dedicated software (Advantage Workstation 4.7 Revolution, GE Healthcare, Milwaukee, WI, USA).

### 2.4. Body Composition Measurements

Skeletal Muscle Mass (SMI) and Low Muscle Mass: At the third lumbar vertebra (L3) level, the total skeletal muscle cross-sectional area (including bilateral psoas, paravertebral, and abdominal wall muscles) was quantified using planimetric segmentation. Skeletal muscle index (SMI, cm^2^/m^2^) was calculated by normalizing total muscle area to height squared. Because muscle strength and physical performance assessments (e.g., handgrip dynamometry) were not available, we limited the assessment to muscle quantity, in line with the EWGSOP2 approach [23]. Sex-specific L3 area and SMI thresholds reported in the literature were used to define low muscle mass: for men, total muscle area < 144.3 cm^2^ and SMI < 45.4 cm^2^/m^2^; for women, total muscle area < 92.2 cm^2^ and SMI < 34.4 cm^2^/m^2^ [24].

Muscle Attenuation and Myosteatosis: Muscle quality was assessed on the same L3 CT slice by measuring mean muscle attenuation (Hounsfield units, HU). Muscle tissue was segmented using a standard attenuation range (−29 to +150 HU), and the mean HU of the entire skeletal muscle area was recorded. Myosteatosis was defined as mean muscle attenuation below sex-specific thresholds (men < 38.5 HU; women < 34.3 HU) [24].

Adipose Tissue: Visceral and subcutaneous adipose tissue areas (VAT and SAT) were measured on the L3 CT slice using an attenuation range of −190 to −30 HU and planimetric calculation [25,26]. Visceral obesity was defined as VAT ≥ 100 cm^2^. In addition, the visceral-to-subcutaneous fat ratio (VSR) was calculated as VAT/SAT, and VSR ≥ 0.73 was used as the threshold for an elevated ratio [27].

### 2.5. Diet Quality Assessment

Dietary habits were evaluated by an experienced dietitian using a face-to-face 24 h dietary recall obtained during annual follow-up visits. Diet quality was scored using the Revised Diet Quality Index (DQI-R) developed by Haines et al. [28]. The DQI-R ranges from 0 to 100, with higher scores reflecting greater adherence to healthy dietary guidelines. Scores were categorized as poor (≤50), needs improvement (51–80), and good (>80). Because no participant had DQI-R > 80, analyses were performed using the poor and needs-improvement categories.

### 2.6. Laboratory and Graft Function Data

Pre-transplant eGFR values and post-transplant serum creatinine and eGFR values (calculated using the CKD-EPI equation, mL/min/1.73 m^2^) at 1, 3, 6, and 12 months, 18 months, and annual follow-up visits were extracted retrospectively [29]. In this study, 18-month and 2-year eGFR values were analyzed as mid-term graft function indicators; early (1–6 months) and later (3–5 years) eGFR values were also reported in group comparisons when available. Serum albumin, 25(OH) vitamin D, and ferritin were recorded as nutrition- and inflammation-related biomarkers. For metabolic profiling, fasting plasma glucose, HbA1c, total cholesterol, HDL cholesterol, LDL cholesterol, and triglyceride levels were collected from medical records. Routine biochemical parameters were measured using Siemens Atellica Solution analyzers (Siemens Healthineers, Erlangen, Germany).

### 2.7. Echocardiographic Assessment

Transthoracic echocardiography was performed for all patients in the left lateral decubitus position, in accordance with the guidelines of the American Society of Echocardiography (ASE) and the European Association of Cardiovascular Imaging (EACVI). Examinations were conducted using a Philips EPIQ series ultrasound system. Two-dimensional imaging, M-mode, color Doppler, and pulsed and continuous wave Doppler modalities were utilized. Left ventricular ejection fraction was calculated using the modified Simpson’s biplane method from apical four- and two-chamber views. Diastolic function was assessed based on mitral E and A wave velocities, E/A ratio, and tissue Doppler-derived e′ velocities. In cases of valvular pathology, flow velocities and pressure gradients were measured using Doppler echocardiography, and, when necessary, pressure gradients were calculated using the modified Bernoulli equation. All measurements were averaged over three consecutive cardiac cycles, or at least five cycles in patients with atrial fibrillation. Values were reported in millimeters (mm) for consistency with international standards.

### 2.8. Statistical Analysis

Statistical analyses were performed using SPSS Statistics 27.0 (IBM Corp., Armonk, NY, USA). Normality was assessed with the Shapiro–Wilk test. Continuous variables are presented as mean ± standard deviation or median (min–max), as appropriate; categorical variables are presented as *n* (%). Group comparisons used Student’s t-test or the Mann–Whitney U test for continuous variables and the chi-square test or Fisher’s exact test for categorical variables. Recipients were compared across subgroups defined by low muscle mass (low vs. normal SMI), myosteatosis (present vs. absent), visceral obesity (high vs. normal VAT), and diet quality category (poor vs. needs improvement). While investigating the associations between non-normally distributed and/or ordinal variables, the correlation coefficients and their significance were calculated Spearman test. A multiple linear regression model was used to identify independent predictors of DQI-R. The model fit was assessed using appropriate residual and goodness-of-fit statistics. Univariable and multivariable logistic regression analyses were performed to identify factors associated with myosteatosis. Candidate variables with *p* < 0.25 in univariable analyses were considered for multivariable modeling, with forward stepwise selection. A two-sided *p* value < 0.05 was considered statistically significant.

## 3. Results

### 3.1. Participant Characteristics

Table 1 presents the baseline demographic, clinical, body composition, dietary, and laboratory characteristics of the study population. A total of 161 KTRs met the inclusion criteria. Mean age was 45.7 ± 13.2 years and 58% were men. The median time since transplantation was 38 months (range, 10–315), and 62% received a living-donor transplant. Median BMI was 24.0 kg/m^2^ (range, 11.1–40.0), and 18.2% (*n* = 28) had obesity by BMI (BMI ≥ 30 kg/m^2^). The prevalence of low muscle mass (low SMI) was 26.0% (*n* = 42). Myosteatosis (low muscle attenuation) was present in 73.5% (*n* = 100 of 136 recipients with available muscle attenuation data). Visceral obesity (VAT ≥ 100 cm^2^) was present in 45.6% (*n* = 62). The median DQI-R score was 52 (range, 29–77), and no participant had DQI-R > 80 (good diet quality).

### 3.2. Comparisons by Diet Quality Category

Table 2 compares clinical, body composition, metabolic, and graft function parameters according to diet quality category. When recipients with poor diet quality (*n* = 83) were compared with those with diet quality that needed improvement (*n* = 78), the groups were similar in age, sex, time since transplantation, and donor type. The median BMI was 26.0 kg/m^2^ (IQR = 6.5) in the needs-improvement group versus 22.1 kg/m^2^ (IQR = 7.24) in the poor group, *p* = 0.005, left ventricular diastolic diameter 45.0 mm (IQR = 0.6) in the needs-improvement group versus 46.0 mm (IQR = 0.6) in the poor group *p* = 0.025, respectively. BMI was significantly higher in the needs-improvement group (median 26.0 vs. 22.1 kg/m^2^; *p* = 0.005). On echocardiography, left ventricular diastolic diameter was slightly higher in the needs-improvement group (median 46 vs. 45 mm; *p* = 0.025), whereas ejection fraction did not differ. CT-derived body composition measures (SMI, muscle attenuation, VAT, VSR, VAT index, and SAT) were comparable between groups (all *p* > 0.05). Metabolic parameters (HbA1c, fasting glucose, lipid profile, albumin, hemoglobin) and eGFR trajectories at different time points were also similar.

### 3.3. Comparisons by Low Skeletal Muscle Index

As shown in Table 3, recipients with low SMI (*n* = 42) had a higher proportion of men than those with normal SMI (73.8% vs. 55.9%; *p* = 0.040). The median BMI was 23.7 kg/m^2^ (IQR = 6.57) in the low SMI group versus 24.9 kg/m^2^ (IQR = 8.45) in the normal SMI group, *p* = 0.018, SAT area 56.0 cm^2^ (IQR = 79.95) in the low SMI group versus 92.1 cm^2^ (IQR = 105.25) in the normal SMI group *p* = 0.006, respectively. BMI was modestly lower in the low SMI group (median 23.7 vs. 24.9 kg/m^2^; *p* = 0.018). Similarly, SAT area was lower among recipients with low SMI (median 56.0 vs. 92.1 cm^2^; *p* = 0.014). Myosteatosis prevalence and DQI-R scores did not differ between groups. Metabolic parameters were also comparable. Although overall eGFR trajectories were similar, the 2-year eGFR value was higher in the low SMI group (74.3 ± 24.0 vs. 56.5 ± 21.8 mL/min/1.73 m^2^; *p* = 0.005).

### 3.4. Comparisons by Myosteatosis Status

Table 4 shows that, among 136 recipients with available muscle attenuation data, those with myosteatosis were significantly older (*p* < 0.001). Sex distribution was similar between groups. While BMI, SMI, and VSR did not differ significantly, VAT area, SAT area, and VAT index were higher in recipients with myosteatosis (all *p* ≤ 0.001). Metabolic markers (glucose indices and lipid profile), diet quality, and eGFR values at different time points did not differ significantly by myosteatosis status.

### 3.5. Correlations Between Diet Quality and Body Composition

As shown in Table 5, DQI-R showed a weak positive correlation with SMI (Spearman r = 0.157; *p* = 0.047). In contrast, DQI-R was not significantly correlated with VAT, SAT, VSR, or muscle attenuation (all *p* > 0.05). Body composition variables were interrelated: VAT was inversely correlated with muscle attenuation (r = −0.335; *p* < 0.001) and positively correlated with SMI (r = 0.248; *p* = 0.004).

### 3.6. Multivariable Analyses

In multivariable linear regression with DQI-R as the dependent variable, the overall model was statistically significant; however, none of the independent variables (age, sex, BMI, SMI, VAT, muscle attenuation, HbA1c, and eGFR) showed a statistically significant association with DQI-R (all *p* > 0.05). In the model predicting HbA1c, the overall model was significant (R^2^ = 0.35), and older age was the strongest predictor (B = 0.038; *p* = 0.002). VAT area was negatively associated with HbA1c (B = −0.001; *p* = 0.004), and DQI-R was positively associated with HbA1c (B = 0.008; *p* = 0.004). The association between muscle attenuation and HbA1c was borderline (*p* = 0.056). In the model predicting eGFR (R^2^ = 0.22), none of the included variables (age, sex, BMI, SMI, VAT, muscle attenuation, DQI-R, and HbA1c) independently predicted eGFR (all *p* > 0.05), although muscle attenuation and DQI-R showed weak positive trends (*p* = 0.054 and *p* = 0.056, respectively). In logistic regression for myosteatosis, older age (OR = 1.10; *p* < 0.001) and lower HbA1c (OR = 0.54; *p* = 0.016) were independently associated with myosteatosis, whereas BMI, SMI, DQI-R, SAT, and eGFR were not.

## 4. Discussion

In this retrospective cohort of KTRs, we evaluated CT-derived body composition measures (muscle quantity, muscle quality, and fat distribution) together with diet quality. Three points stand out. First, myosteatosis was common and often clustered with visceral adiposity. Second, diet quality was generally suboptimal, suggesting that post-transplant nutrition should not focus only on calories or body weight but also on dietary pattern quality [4,21,23]. Third, because creatinine-based eGFR can be biased in the presence of low muscle mass, graft function should be interpreted together with body composition and, when possible, supported by cystatin C (and/or creatinine–cystatin C combined equations) or measured GFR [25,26,27]. Overall, these findings support a more integrated interpretation of “body composition” that considers muscle quantity, muscle quality, fat distribution, and diet quality together.

### 4.1. Myosteatosis and Muscle Quality

Myosteatosis was frequently identified in our cohort based on muscle attenuation. Myosteatosis reflects impaired muscle quality related to intramuscular lipid deposition. It should not be viewed as an isolated “muscle” finding. It is better interpreted together with ectopic fat deposition and metabolic phenotype [16]. The older age of recipients with myosteatosis is consistent with the concept that intramuscular fat infiltration increases with age [17]. Prior studies also suggest that myosteatosis may signal clinical risk independent of low muscle mass [18,19]. Therefore, it is reasonable to consider myosteatosis as part of a broader clinical risk phenotype rather than a minor radiologic observation.

In our data, myosteatosis clustered not only with age but also with fat distribution. Recipients with myosteatosis had higher VAT area, VAT index, and SAT area. This pattern suggests that myosteatosis may represent a composite phenotype related to ectopic fat deposition and adiposity distribution, rather than “muscle loss alone”. Similar associations have been reported in CKD and post-transplant populations using both CT-based and MRI-based imaging, supporting the external consistency of our findings [17,28]. Because visceral and subcutaneous fat depots have different biology, and because visceral fat can predict post-transplant diabetes risk, reporting myosteatosis together with fat distribution measures may strengthen clinical interpretation [20,22].

The negative correlation between VAT and muscle attenuation further supports the co-occurrence of visceral fat expansion and reduced muscle quality. This supports the view that risk assessment should go beyond BMI and should incorporate both fat distribution and muscle quality [28,29]. Olcucuoglu et al. also highlighted that muscle quality metrics may provide a stronger risk signal than low muscle mass for certain transplant outcomes [28].

The absence of a clear difference in eGFR trajectories between myosteatosis groups may have two explanations. First, creatinine-based eGFR can interact with muscle composition. Second, the impact of myosteatosis may become more visible through metabolic complications and longer-term outcomes before it translates into detectable eGFR differences [18,19]. Therefore, in post-transplant follow-up, myosteatosis may be better positioned as a body composition abnormality related to metabolic risk clustering. This view aligns with prior transplant studies showing myosteatosis to be a stronger predictor of cardiovascular risk than SMI alone, especially when paired with markers of inflammation and insulin resistance [19,28].

### 4.2. Diet Quality

The lack of any participant with “good” diet quality and the overall suboptimal DQI-R distribution indicate a clear area for improvement in post-transplant nutritional care [4,21,23]. Reduced dialysis-era restrictions, increased appetite, and the metabolic effects of immunosuppression may contribute to this pattern [3,4]. These findings support the need for sustained dietitian involvement during post-transplant follow-up [23]. Although few studies have evaluated DQI-R specifically in transplant recipients, our results align with broader evidence suggesting poor adherence to dietary recommendations after transplantation [4,21]. Moreover, compared to studies using Mediterranean Diet Scores or Healthy Eating Index (HEI), our use of DQI-R provides a broader perspective on macronutrient distribution, though it may be less sensitive to transplant-specific dietary nuances [30,31].

Diet quality is a modifiable lifestyle factor. However, in this cohort, its association with CT-based body composition measures was not strong. This should not be interpreted as “no effect”. Rather, it likely reflects the limitations of retrospective design, single-time-point diet assessment, and unmeasured or heterogeneous confounding (e.g., physical activity and treatment-related factors).

The higher BMI observed in the needs-improvement diet group suggests that the relationship between diet quality and body weight is not necessarily linear. Reverse causality is possible; recipients followed more closely due to weight or metabolic risk may adopt relatively better dietary choices. Reported associations between diet quality and obesity can vary by population and measurement approach [32]. Moreover, diet quality derived from a single-day record may not fully capture long-term dietary patterns. As a result, group differences in slowly changing outcomes such as body composition may be attenuated [33]. In this context, the lack of differences in SMI, muscle attenuation, VAT, and fat indices across diet-quality groups is not unexpected. Physical activity is likely a key co-determinant, and prospective data support its relationship with post-transplant body composition [24]. Still, the weak but significant positive correlation between DQI-R and SMI suggests a limited signal linking better diet quality to higher muscle mass [34].

### 4.3. Low Muscle Mass

Low SMI was present in approximately one-quarter of recipients, consistent with prior reports [8,35]. Because we lacked muscle strength and performance measurements, we used the term “low muscle mass/low SMI” rather than “sarcopenia”, which is more consistent with the EWGSOP2 framework [34].

The higher proportion of men in the low SMI group suggests that sex-related factors may influence body composition phenotypes. This observation may also reflect sex-specific threshold issues described in the literature [36]. In addition, the lower SAT area in the low SMI group may point to a distinct phenotype with lower fat reserves.

The higher 2-year creatinine-based eGFR in recipients with low SMI is clinically important. In low muscle mass, creatinine generation is reduced, which can lead to spuriously higher eGFR estimates [27]. This supports the view that muscle composition can bias creatinine-based assessment [37]. Accordingly, when clinical decisions depend on eGFR, it may be appropriate to corroborate creatinine-based eGFR with markers less affected by muscle mass (e.g., cystatin C or combined equations) or with measured GFR [26]. Our findings are consistent with previous studies demonstrating a discordance between low muscle mass and elevated eGFR values when creatinine-based formulas are used in patients with reduced muscle mass [37]. Low muscle mass may also be linked to patient-centered outcomes such as mortality, underscoring that creatinine-based eGFR may not fully capture overall risk [26,38].

### 4.4. Visceral Adiposity

Visceral adiposity is a recognized contributor to post-transplant metabolic risk, and CT-derived measures of visceral fat have been shown to predict outcomes such as post-transplant diabetes [20]. In our cohort, visceral fat measures were higher in recipients with myosteatosis, reinforcing the idea that risk may cluster across compartments. At the same time, the lack of clear separation in creatinine-based eGFR trajectories across VAT or diet-quality groups may reflect the multifactorial determinants of graft function and the measurement characteristics of eGFR [27]. Taken together with the work by Olcucuoglu et al., our findings support evaluating muscle quality and visceral adiposity together to better characterize post-transplant risk phenotypes [28]. This integrated approach mirrors recent efforts in transplant medicine to move beyond BMI and adopt body composition-driven phenotyping for personalized metabolic risk stratification [28,38].

### 4.5. Clinical Implications and Integrated Multidisciplinary Care

The findings of this study should be interpreted within contemporary, integrated kidney care models that extend beyond graft survival toward long-term metabolic and functional outcomes. As KTRs age, nutritional status, body composition, and cardiometabolic risk increasingly shape long-term health. Although diet quality was not independently associated with short- to medium-term creatinine-based eGFR, the overall suboptimal dietary patterns observed underscore the need for sustained nutritional counseling. Diet quality should be considered not only in relation to body weight, but also as a modifiable factor influencing metabolic health and muscle preservation.

Muscle quality, particularly myosteatosis, emerged as a key component of integrated care, frequently clustering with visceral adiposity. This finding supports evaluating muscle and fat compartments together rather than in isolation, as these phenotypes may develop early and precede overt graft dysfunction. Clinically, these results support structured, multidisciplinary follow-up involving nephrologists, transplant nurses, and dietitians. Post-transplant monitoring may therefore move beyond a “BMI-only” approach toward integrated assessment of diet quality and CT-derived body composition to enable more personalized long-term management.

### 4.6. Limitations and Strengths

This study has several limitations. Its retrospective, single-center design limits causal inference and may reduce generalizability to other transplant populations with different clinical practices or immunosuppressive protocols; therefore, findings should be interpreted as descriptive. Although CT-derived measures enabled objective body composition assessment, muscle attenuation data were unavailable for all recipients, introducing potential selection bias and rendering myosteatosis-related results hypothesis-generating. In addition, the absence of muscle strength and physical performance assessments precluded a comprehensive evaluation of sarcopenia. Diet quality was assessed at a single time point using self-reported data, which may not reflect long-term dietary patterns, and residual confounding may persist due to unmeasured factors such as physical activity and treatment-related variables. Finally, variability in CT acquisition and reconstruction protocols may have influenced muscle attenuation measurements.

The strengths of this study include its detailed and multidimensional evaluation of body composition in KTRs, an area that remains underexplored. The use of CT-derived objective measures (SMI, muscle attenuation, and VAT) provided a robust framework for body phenotyping. Integration of diet quality assessment with metabolic and longitudinal graft function data, along with a real-world heterogeneous cohort, enhances the clinical relevance and applicability of the findings.

## 5. Conclusions

In KTRs myosteatosis represents an important body composition feature that frequently coexists with visceral adiposity, indicating that muscle quality and fat distribution should be evaluated together. The generally suboptimal diet quality and its limited association with muscle mass suggest that nutritional assessment should not focus solely on body weight. These findings emphasize that post-transplant follow-up should move beyond a graft function-centered approach toward a multidisciplinary care model that incorporates body composition and nutritional status.

## Figures and Tables

**Table 1:** Baseline demographic, body composition, diet, laboratory, and graft function characteristics of KTRs (*n* = 161).

Parameter	Value
Age (years)	45.7 ± 13.2
Sex (male/female, %)	58/42
Time since transplantation (months)	38 (10–315)
Donor type (living/deceased, %)	62/38
**Body composition**	
BMI (kg/m^2^)	24.0 (11.1–40.0)
Obesity by BMI (≥30 kg/m^2^)	18.2% (*n* = 28)
Skeletal muscle index (SMI, cm^2^/m^2^)	42.4 ± 11.2
Low SMI (%)	26.0% (*n* = 42)
Muscle attenuation (HU)	31.7 ± 8.7
Myosteatosis present (%)	73.5% (*n* = 100)
Visceral adipose tissue area (VAT, cm^2^)	89.0 (3.2–352.1)
Visceral obesity (VAT ≥ 100 cm^2^, %)	45.6% (*n* = 62)
VSR	1.1 ± 0.6
High VSR (%)	66.9% (*n* = 91)
VAT index	30.6 (1.5–103.8)
High VAT index (%)	48.0% (*n* = 60)
SAT area (SAT, cm^2^)	83.3 (6.4–514.4)
High SAT (%)	22.1% (*n* = 30)
**Diet characteristics**	
DQI	52 (29–77)
DQI category (poor/needs improvement, %)	48.4 (*n* = 78)/51.6 (*n* = 83)
**Laboratory parameters**	
Albumin (g/L)	44.2 (14.1–74.0) (*n* = 158)
HbA1c (%)	5.6 (4.1–13.2) (*n* = 123)
Fasting glucose (mg/dL)	95.0 (69.0–806.0) (*n* = 129)
LDL cholesterol (mg/dL)	96.3 (22.0–686.0) (*n* = 148)
HDL cholesterol (mg/dL)	46.0 (23.0–103.0) (*n* = 147)
Triglycerides (mg/dL)	140.0 (42.0–600.0) (*n* = 145)
Serum creatinine (mg/dL)	1.3 (0.2–14.0) (*n* = 147)
**Graft function (eGFR, mL/min/1.73 m^2^)**	
eGFR, 1 month (mL/min/1.73 m^2^)	55.0 ± 22.0
eGFR, 3 months	60.0 ± 20.0
eGFR, 6 months	63.0 ± 21.0
eGFR, 12 months	65.5 ± 21.0
eGFR, 2 years	62.5 ± 24.6

Continuous variables are presented as mean ± SD or median (min–max), as appropriate. Abbreviations: BMI, body mass index; DQI, Diet Quality Index; eGFR, estimated glomerular filtration rate; HU, Hounsfield unit; SAT, subcutaneous adipose tissue; SMI, skeletal muscle index; VAT, visceral adipose tissue; VSR, visceral-to-subcutaneous fat ratio.

**Table 2:** Comparison of clinical, body composition, laboratory, and eGFR time-series parameters according to diet quality category.

Parameter	Poor DQI (*n* = 83)	Needs-Improvement DQI (*n* = 78)	*p* Value
Age (years)	45.5 ± 14.6	46.7 ± 11.6	0.565
Male (%)	57.8	59.0	0.883
Time since transplantation (months)	40 (10–315)	36 (13–261)	0.772
Living donor (%)	60.6	64.2	0.661
BMI (kg/m^2^)	22.1 (16.6–40.0)	26.0 (11.1–39.0)	0.005 *
SMI (cm^2^/m^2^)	50.7 ± 9.3	51.9 ± 9.9	0.494
Muscle attenuation (HU)	31.4 (7.7–46.7)	32.6 (13.2–50.3)	0.685
VAT area (cm^2^)	125 (10–300)	140 (15–290)	0.251
VSR	0.9 (0.2–3.5)	1.0 (0.2–3.2)	0.789
VAT index	26.5 (1.5–83.7)	34.1 (6.6–103.8)	0.150
SAT area (cm^2^)	75.7 (7.0–514.4)	110.0 (7.4–381.0)	0.239
Left ventricular diameter mm	45 (33–63)	46 (39–64)	0.025 *
Ejection fraction (%)	60 (40–66)	60 (25–65)	>0.05
HbA1c (%)	5.7	5.6	>0.05
Fasting glucose (mg/dL)	92	95	>0.05
LDL cholesterol (mg/dL)	98	96	0.937
Triglycerides (mg/dL)	140	140.5	0.573
Albumin (g/L)	44.2	44.1	0.955
Hemoglobin (g/dL)	12.9 ± 1.4	13.1 ± 1.3	0.893
eGFR, 1 month (mL/min/1.73 m^2^)	55 ± 22	53 ± 23	0.472
eGFR, 3 months	60 ± 20	58 ± 24	0.516
eGFR, 6 months	63 ± 21	61 ± 25	0.553
eGFR, 12 months	65.5 ± 21.0	62.3 ± 25.5	0.498
eGFR, 2 years	62.5 ± 24.6	59.0 ± 23.1	0.546

*: *p* value by Mann–Whitney U test. Continuous variables are presented as mean ± SD or median (min–max), as appropriate. Abbreviations: BMI, body mass index; DQI, Diet Quality Index; eGFR, estimated glomerular filtration rate; HU, Hounsfield unit; SAT, subcutaneous adipose tissue; SMI, skeletal muscle index; VAT, visceral adipose tissue; VSR, visceral-to-subcutaneous fat ratio.

**Table 3:** Comparison of clinical, body composition, metabolic, and longitudinal eGFR parameters according to SMI status.

Parameter	Low SMI (*n* = 42, 26.1%)	Normal SMI (*n* = 119, 73.9%)	*p* Value
Age (years)	47.1 ± 13.0	45.4 ± 13.3	0.475
Male (%)	73.8 (*n* = 42)	55.9 (*n* = 42)	0.040 ***
BMI (kg/m^2^)	23.7 (17.0–40.0)	24.9 (11.1–39.0)	0.018 *
Myosteatosis (HU)	32.8 ± 10.1	32.5 ± 8.7	0.816
VAT area (cm^2^)	81.7 (3.2–259.0)	89.1 (10.3–352.1)	0.194
VSR	1.2 (0.3–3.2)	0.9 (0.2–3.5)	0.335
VAT index	30.6 (1.5–103.8)	30.3 (4.3–98.5)	0.458
SAT area (cm^2^)	56.0 (6.4–381.0)	92.1 (7.4–514.4)	0.014 *
DQI score	50.7 ± 9.3	52.0 ± 9.8	0.453
HbA1c (%)	5.8 (4.1–8.5)	5.6 (4.4–13.2)	0.385
Fasting glucose (mg/dL)	92 (75–461)	95 (69–806)	0.526
LDL cholesterol (mg/dL)	98.4 (22.0–176.4)	96.3 (40.4–686.0)	0.936
HDL cholesterol (mg/dL)	48.0 (28.0–103.0)	46.0 (23.0–78.0)	0.161
Triglycerides (mg/dL)	140.0 (50.0–260.0)	141.0 (42.0–60.0)	0.519
eGFR, 1. ay	58.3 ± 27.6	54.6 ± 25.6	0.496
eGFR, 3 months	64.1 ± 26.9	63.2 ± 23.4	0.864
eGFR, 6 months	67.8 ± 27.3	63.4 ± 22.5	0.400
eGFR, 12 months	70.2 ± 23.1	62.8 ± 23.7	0.161
eGFR, 2 years	74.3 ± 24.0	56.5 ± 21.8	0.005 **

*: *p* value by Mann–Whitney U test, **: *p* value by Student’s test and ***: *p* value by Chi-square test. Continuous variables are presented as mean ± SD or median (min–max), as appropriate. Abbreviations: BMI, body mass index; DQI, Diet Quality Index; eGFR, estimated glomerular filtration rate; HU, Hounsfield unit; SAT, subcutaneous adipose tissue; SMI, skeletal muscle index; VAT, visceral adipose tissue; VSR, visceral-to-subcutaneous fat ratio.

**Table 4:** Comparison of clinical, body composition, and metabolic parameters according to myosteatosis status in participants with available muscle attenuation data (*n* = 136).

Parameter	Myosteatosis Present (*n* = 100)	Myosteatosis Absent (*n* = 36)	*p* Value
Age (years)	46.2 ± 12.0	35.9 ± 11.2	<0.001 **
Male (%)	62.0 (*n* = 62)	61.1 (*n* = 22)	0.925
BMI (kg/m^2^)	24.7 (11.1–39.0)	22.0 (18.0–39.0)	0.190
SMI (cm^2^/m^2^)	42.6 ± 11.3	42.8 ± 10.2	0.816
Muscle attenuation (HU)	29.8 (7.7–38.4)	41.1 (35.5–50.3)	<0.001 *
VAT area (cm^2^)	109.1 (3.2–352.1)	57.7 (10.3–158.6)	<0.001 *
SAT area (cm^2^)	107.8 (6.4–514.4)	54.7 (7.4–264.5)	0.001 *
VSR	1.0 (0.2–3.5)	0.8 (0.3–2.4)	0.163
VAT index	37.7 (1.5–103.8)	30.3 (4.3–58.3)	<0.001 *
DQI score	52.0 (29.0–77.0)	52.0 (35.0–70.0)	0.690
HbA1c (%)	5.6 (4.4–8.5)	5.4 (4.1–13.2)	0.407
Fasting glucose (mg/dL)	94.5 (69.0–461.0)	95.0 (76.0–806.0)	0.523
LDL cholesterol (mg/dL)	99.3 (22.0–202.0)	92.4 (55.0–686.0)	0.741
HDL cholesterol (mg/dL)	45.7 ± 14.3	48.0 ± 13.2	0.430
Triglycerides (mg/dL)	139.0 (50.0–600.0)	142.5 (42.0–458.0)	0.560
eGFR, 1. ay	53.4 ± 25.3	60.2 ± 32.3	0.244
eGFR, 3 months	62.2 ± 24.2	68.2 ± 27.4	0.269
eGFR, 6 months	63.8 ± 22.6	67.6 ± 28.6	0.477
eGFR, 12 months	64.6 ± 22.8	70.8 ± 28.7	0.287
eGFR, 2 years	63.5 ± 23.2	56.2 ± 26.6	0.308

*: *p* value by Mann–Whitney U test and **: *p* value by Student’s test Continuous variables are presented as mean ± SD or median (min–max), as appropriate. Abbreviations: BMI, body mass index; DQI, Diet Quality Index; eGFR, estimated glomerular filtration rate; HbA1c, glycated hemoglobin; HDL, high-density lipoprotein; LDL, low-density lipoprotein; SAT, subcutaneous adipose tissue; SMI, skeletal muscle index; VAT, visceral adipose tissue; VSR, visceral-to-subcutaneous fat ratio.

**Table 5:** Spearman correlation analysis between DQI-R and CT-derived body composition parameters.

Variables	r	*p*
DQI-R vs. VAT	0.100	0.248
DQI-R vs. SAT	0.110	0.224
DQI-R vs. VSR	0.010	0.884
DQI-R vs. SMI	0.157	0.047 *
DQI-R vs. myosteatosis (muscle attenuation)	0.013	0.884
VAT vs. SMI	0.248	0.004 *
VAT vs. myosteatosis (muscle attenuation)	−0.335	<0.001 *
SMI vs. myosteatosis (muscle attenuation)	0.350	0.694

*: *p* value by Spearman test. Abbreviations: DQI-R, Revised Diet Quality Index; SAT, subcutaneous adipose tissue; SMI, skeletal muscle index; VAT, visceral adipose tissue; VSR, visceral-to-subcutaneous fat ratio. r indicates correlation coefficient and *p* indicates significance level.

## Data Availability

The data presented in this study are available from the corresponding author upon reasonable request, subject to institutional and privacy restrictions.

## Abbreviations

CT	Computed tomography
DQI-R	Revised Diet Quality Index
VSR	Visceral-to-subcutaneous adipose tissue ratio
SMI	Skeletal muscle index
SMA	Skeletal muscle area
KTRs	Kidney transplant recipients
BMI	Body mass index
HU	Hounsfield unit
DM	Diabetes mellitus
HT	Hypertension
SD	Standard deviation
IQR	Interquartile range
eGFR	Estimated glomerular filtration rate
MS	Metabolic syndrome
VAT	Visceral adipose tissue
